# Relationship between brachial-ankle and heart-femoral pulse wave velocities and the rapid decline of kidney function

**DOI:** 10.1038/s41598-018-19334-w

**Published:** 2018-01-16

**Authors:** Sung Woo Lee, Seung Hyeok Han, Tae Hyun Yoo, Wookyung Chung, Sue K. Park, Dong Wan Chae, Curie Ahn, Kook-Hwan Oh

**Affiliations:** 1 Department of Internal Medicine, Seoul National University Postgraduate School, Seoul, Korea; 2 Department of Internal Medicine, Eulji University, Eulji General Hospital, Seoul, Korea; 3 Department of Internal Medicine, Yonsei University College of Medicine, Seoul, Korea; 4 Department of Internal Medicine, Gachon University, Gil Hospital, Incheon, Korea; 5 Department of Preventive Medicine, Seoul National University College of Medicine, Seoul, Korea; 6 Cancer Research Institute, Seoul National University, Seoul, Korea; 7 Department of Biomedical Science, Seoul National University Graduate School, Seoul, Korea; 8 Department of Internal Medicine, Seoul National University College of Medicine, Seoul, Korea

## Abstract

The impact of brachial-ankle pulse wave velocity (baPWV) and heart-femoral pulse wave velocity (hfPWV) on rapid decline of estimated glomerular filtration rate (eGFR) has been inconclusive. The database of a multicenter prospective study of 2238 patients in Korea enrolled from 2011 to 2016 was reviewed. After excluding patients with missing baPWV (n = 257) and eGFR change (n = 180), the study included 1801 non-dialysis chronic kidney disease (CKD) patients. The eGFR change <−5ml/min/1.73 m^2^/year was defined as rapid decline. During a mean of 2.2 years, the mean eGFR change was −3.6 ml/min/1.73 m^2^/year, and 31.6% of patients were classified as having rapid decline. Older age, causes of CKD, increased heart rate, systolic blood pressures, and proteinuria were associated with the highest baPWV quintile. In multivariate logistic regression analyses, the odds of a rapid decline in eGFR was 1.9 times higher in the fifth quintile than in the first quintile (*P* = 0.013). In a subset with baPWV and hfPWV (n = 1182), high baPWV was associated with rapid eGFR decline only when accompanied by a high hfPWV. These findings suggest that central and peripheral PWVs may simultaneously affect rapid eGFR decline.

## Introduction

Chronic kidney disease (CKD) is a common form of chronic disease^1^, and it contributes to an increased risk of the development of cardiovascular (CV) events and mortality^2^. As CKD progresses, decreased kidney function can increase arterial stiffness^3^. Conversely, increased arterial stiffness can lead to deterioration of kidney function^4^. Whether there is a bidirectional association between increased arterial stiffness and decreased kidney function is unclear, but a recent study suggested that increased arterial stiffness affected the decline of kidney function uni-directionally^5^. This can be explained by the fact that increased arterial stiffness limits the dampening of ventricular ejection which transmits more pressure to the end organs^6^, and the increased pressure that is transmitted to the low-resistance renal afferent arterioles can damage the glomerular capillaries, resulting in reduced kidney function^7^.

The standard method to evaluate arterial stiffness is measuring the pulse wave velocity (PWV)^8^, and the most validated PWV is carotid-femoral PWV (cfPWV), but its measurement requires expert techniques^9^. For more convenient and reproducible results, a device has been developed that uses an oscillometric method to record the pulse waves of the brachial and posterior tibial arteries, and it can calculate the brachial-ankle PWV (baPWV)^10^. The baPWV correlates well with cfPWV^11^, and evidence has shown that the baPWV is also a good marker of arterial stiffness^12,13^.

Until now, many studies have suggested that increased arterial stiffness is associated with an increased risk of kidney function decline^4,5,14–16^. However, different results have also been reported^17–20^. In addition, despite one Taiwanese group’s extensive efforts^21–24^, studies using the baPWV to predict the decline of kidney function have been insufficient, and their results were also inconclusive due to small patient numbers from single centers^25–29^. Therefore, we performed the current multicenter study to identify the clinical importance of the baPWV on the decline in kidney function using data on a large number of adults who were enrolled in the KoreaN cohort study for Outcome in patients With Chronic Kidney Disease (KNOW-CKD).

## Results

There were 1801 patients in this study, of which 60.6% were men. The mean age of all patients was 54.0 years. The causes of CKD were diabetic nephropathy in 24.7% of patients, glomerulonephritis in 32.6%, hypertensive nephropathy in 19.9%, and other causes in 22.9%. At enrollment, the mean baseline estimated glomerular filtration rate (eGFR) and median urine protein-to-creatinine ratio (UPCR) were 51.7 ml/min/1.73 m^2^ and 0.5 g/g creatinine, respectively. The mean ΔeGFR was −3.6 ml/min/1.73 m^2^/year, and 31.6% of the participants were classified as having a rapid decline in the eGFR. Of 1801 patients, data on dialysis initiation and CV events were available for 1784 (99.1%). During a mean of 2.4 years, 225 (12.5%) initiated dialysis and 74 (4.1%) developed CV events.

We compared the baseline characteristics of the patients according to the quintile of baPWV (Table 1). Older age was associated with increased baPWV. The percentages of diabetic nephropathy and hypertensive nephropathy increased and those of glomerulonephritis and other causes of CKD decreased with the increase of baPWV. As the quintile of baPWV increased, smoking pack-year, use of beta blockers, calcium channel blockers, diuretics, rate of CV disease, pulse pressure, and heart rate increased. Bilirubin, albumin, hemoglobin level, and eGFR decreased, but high-sensitivity C-reactive protein (hsCRP) and UPCR increased as the baPWV increased. With the progression of baPWV, serum calcium levels decreased, whereas serum phosphorus levels increased. To adjust for confounders, we performed a multivariate logistic regression analysis (Table 2), and found that patients’ age, causes of CKD, heart rate, systolic BP, calcium channel blockers use, serum levels of calcium, hsCRP, and UPCR were independently associated with the highest quintile of baPWV. With the progression of CKD stages, age, smoking pack-year, rates of hypertension, diabetes, CV disease, use of beta blockers, calcium channel blockers, diuretics, pulse pressure, PWVs, serum levels of phosphorus, hsCRP, and UPCR increased, but alcohol drinking, serum levels of calcium, bilirubin, albumin, and hemoglobin decreased (Table S1).

**Table 1:** Baseline characteristics of patients according to the status of baPWV.

Total (n = 1801)	Quintile of baPWV					*P*-trend
	1Q (n = 370)	2Q (n = 346)	3Q (n = 354)	4Q (n = 373)	5Q (n = 358)	
Age (years)	44.3 ± 11.4	49.7 ± 10.8*	53.5 ± 10.7*	58.9 ± 9.2*	63.5 ± 8.2*	<0.001
Male sex (%)	57.3	60.7	59.9	62.7	62.3	0.134
Alcohol drinking (%)	49.7	48.6	47.4	36.9*	40.8*	0.001
Smoking (Pack-year)	7.2 ± 13.4	10.8 ± 17.6	10.7 ± 16.7	14.1 ± 19.6*	16.2 ± 22.8*	<0.001
Hypertension (%)	92.2	96.5*	96.0*	96.0*	97.5*	0.003
RAS inhibitor (%)	86.2	87.0	89.3	84.4	87.7	0.951
Beta blocker (%)	17.0	21.1	23.7*	29.8*	30.7*	<0.001
CCB (%)	25.9	37.6*	44.9*	48.7*	58.7*	<0.001
Diuretics (%)	18.6	21.4	26.8*	43.0*	43.0*	<0.001
Diabetes (%)	6.8	12.5*	19.9*	39.2*	52.8*	<0.001
CVD (%)	4.3	7.8	9.6*	12.9*	16.5*	<0.001
Cause of CKD						
DMN (%)	5.1	11.0*	15.0*	37.0*	54.7*	<0.001
HN (%)	12.4	17.3	25.4*	24.4*	19.8*	0.001
GN (%)	53.8	43.9*	30.5*	22.5*	12.3*	<0.001
Others (%)	28.6	27.7	29.1	16.1*	13.1*	<0.001
SBP (mmHg)	120.3 ± 13.5	124.8 ± 13.7*	129.0 ± 14.2*	130.7 ± 16.2*	137.5 ± 17.3*	<0.001
DBP (mmHg)	74.2 ± 10.0	76.8 ± 10.8*	78.7 ± 10.4*	77.3 ± 12.0*	77.5 ± 11.6*	<0.001
PP (mmHg)	46.2 ± 10.3	48.0 ± 10.8	50.3 ± 11.5*	53.4 ± 12.2*	60.0 ± 13.8*	<0.001
HR (counts/min)	71.9 ± 12.7	72.1 ± 12.6	73.1 ± 13.0	73.4 ± 12.7	75.7 ± 13.0*	<0.001
baPWV (m/s)	11.6 ± 0.8	13.3 ± 0.4*	14.6 ± 0.4*	16.5 ± 0.7*	20.5 ± 2.8*	<0.001
hfPWV (m/s)	7.8 ± 1.3	8.9 ± 1.3*	9.7 ± 1.7*	11.3 ± 2.1*	13.0 ± 2.9*	<0.001
BMI (kg/m^2^)	24.3 ± 3.7	24.4 ± 3.8	24.4 ± 3.4	24.6 ± 3.0	24.8 ± 3.0	0.040
FPG (mmol/l)	5.5 ± 1.2	5.8 ± 1.9	6.0 ± 1.7*	6.5 ± 2.5*	6.9 ± 2.8*	<0.001
BUN (mmol/l)	7.8 ± 4.6	8.4 ± 4.3	9.7 ± 5.3*	11.2 ± 5.4*	11.5 ± 5.5*	<0.001
Calcium (mmol/l)	2.3 ± 0.11	2.3 ± 0.12	2.29 ± 0.12	2.28 ± 0.13	2.26 ± 0.14*	<0.001
Phosphorus (mmol/l)	1.15 ± 0.19	1.15 ± 0.19	1.18 ± 0.2	1.2 ± 0.19*	1.23 ± 0.25*	<0.001
Cr (μmol/l)	127.3 ± 79.9	137.5 ± 79.5	149.2 ± 83.7*	173.6 ± 89.7*	177.3 ± 87.1*	<0.001
eGFR (ml/min/1.73 m^2^)	65.9 ± 34.6	59.1 ± 31.8*	52.4 ± 29.9*	42.3 ± 23.3*	39.2 ± 20.7*	<0.001
Bilirubin (μmol/l)	13.1 ± 5.8	12.5 ± 5.7	12.1 ± 4.8	10.4 ± 4.0*	9.9 ± 4.3*	<0.001
Albumin (g/l)	42.7 ± 3.6	42.2 ± 4.2	42.3 ± 3.4	41.6 ± 4.6*	40.9 ± 4.5*	<0.001
Cholesterol (mmol/l)	4.6 ± 0.9	4.5 ± 0.9	4.7 ± 1.0	4.4 ± 1.1	4.5 ± 1.0	0.024
Hemoglobin (g/dl)	13.4 ± 1.9	13.3 ± 2.0	13.1 ± 1.9	12.4 ± 2.0*	12.1 ± 2.0*	<0.001
hsCRP (nmol/l)	4.8 (1.9–13.5)	4.8 (1.9–13.3)	5.7 (1.9–13.3)	6.7 (2.9–17.8)*	8.6 (2.9–18.3)*	<0.001
UPCR (g/g Cr)	0.3 (0.1–0.8)	0.3 (0.1–0.9)	0.5 (0.1–1.3)*	0.6 (0.2–2.2)*	0.9 (0.3–2.7)*	<0.001

RAS, renin angiotensin-aldosterone system; CCB, calcium channel blocker; CVD, cardiovascular disease; baPWV, brachial-ankle pulse wave velocity; CKD, chronic kidney disease; DMN, diabetic nephropathy; HN, hypertensive nephropathy; GN, glomerulonephritis; SBP, systolic blood pressure; DBP, diastolic blood pressure; PP, pulse pressure; hfPWV, heart-femoral pulse wave velocity; BMI, body mass index; FPG, fasting plasma glucose; BUN, blood urea nitrogen; Cr, creatinine; eGFR, estimated glomerular filtration rate; hsCRP, high sensitivity C-reactive protein; UPCR, urine protein-to-creatinine ratio.

Values are expressed as mean ± standard deviation for normally distributed continuous variables, median (interquartile range) for non-normally distributed continuous variables, and percentage for categorical variables. *P*-trend was analyzed by linear-term of one-way ANOVA for normally distributed continuous variables, Jonckheere-Terpstra test for non-normally distributed continuous variables, and a linear-by-linear association for categorical variables. Except for hfPWV (619/1801, 34.4%) and smoking year (286/1801, 15.9%), missing rate of all above variables was below 8.9%.

*Meant *P* < 0.05 when compared to 1Q of baPWV group by using Bonferroni post-hoc analysis of one-way ANOVA for normally distributed continuous variables, Mann-Whitney U test for non-normally distributed continuous variables, and chi-square test for categorical variables.

**Table 2:** Factors associated with highest quintile of baPWV.

	Univariate		Multivariate	
	OR (95% CI)	*P*	OR (95% CI)	*P*
Age (years)	1.120 (1.103–1.136)	<0.001	1.131 (1.108–1.155)	<0.001
Men (vs. women)	1.094 (0.862–1.389)	0.459	0.655 (0.413–1.040)	0.073
Causes of CKD				
HN vs. DMN	0.313 (0.227–0.431)	<0.001	0.506 (0.317–0.808)	0.004
GN vs. DMN	0.103 (0.072–0.147)	<0.001	0.297 (0.179–0.493)	<0.001
Others vs. DMN	0.163 (0.114–0.233)	<0.001	0.572 (0.328–0.996)	0.049
SBP (mmHg)	1.044 (1.036–1.052)	<0.001	1.036 (1.025–1.047)	<0.001
DBP (mmHg)	1.006 (0.996–1.017)	0.227	—	—
RASI (yes vs. no)	1.096 (0.772–1.556)	0.607	—	—
BB (yes vs. no)	1.489 (1.153–1.923)	0.002	1.196 (0.822–1.739)	0.350
CCB (yes vs. no)	2.196 (1.735–2.779)	<0.001	1.570 (1.107–2.227)	0.011
Diuretics (yes vs. no)	1.980 (1.559–2.515)	<0.001	0.911 (0.638–1.302)	0.610
CVD (yes vs. no)	2.081 (1.490–2.906)	<0.001	0.725 (0.447–1.175)	0.192
BMI (kg/m^2^)	1.033 (0.999–1.069)	0.059	—	—
Smoking (Pack-year)	1.014 (1.008–1.020)	<0.001	1.005 (0.996–1.015)	0.246
HR (counts/min)	1.018 (1.010–1.027)	<0.001	1.027 (1.013–1.040)	<0.001
FPG (mmol/l)	1.187 (1.131–1.246)	<0.001	1.007 (0.941–1.077)	0.847
BUN (mmol/l)	1.072 (1.051–1.094)	<0.001	0.971 (0.926–1.017)	0.216
eGFR (ml/min/1.73 m^2^)	0.978 (0.973–0.983)	<0.001	0.998 (0.988–1.008)	0.712
Calcium (mmol/l)	0.148 (0.060–0.368)	<0.001	0.198 (0.041–0.962)	0.045
Phosphorus (mmol/l)	4.046 (2.318–7.061)	<0.001	1.623 (0.635–4.149)	0.312
Bilirubin (μmol/l)	0.902 (0.876–0.929)	<0.001	0.996 (0.949–1.045)	0.870
Albumin (g/l)	0.933 (0.909–0.958)	<0.001	1.044 (0.990–1.100)	0.111
Cholesterol (mmol/l)	0.922 (0.820–1.038)	0.182	0.976 (0.865–1.101)	0.692
Hemoglobin (g/dl)	0.783 (0.736–0.833)	<0.001	1.075 (0.954–1.212)	0.237
Log hsCRP (nmol/l)	1.162 (1.067–1.265)	0.001	1.272 (1.089–1.485)	0.002
Log UPCR (g/g Cr)	1.439 (1.323–1.565)	<0.001	1.131 (1.108–1.155)	<0.001

baPWV, brachial-ankle pulse wave velocity; OR, odds ratio; CI, confidence interval; CKD, chronic kidney disease; DMN, diabetic nephropathy; HN, hypertensive nephropathy; GN, glomerulonephritis; SBP, systolic blood pressure; DBP, diastolic blood pressure; RASI, renin angiotensin-aldosterone system inhibitor; BB, beta blocker; CCB, calcium channel blocker; BMI, body mass index; HR, heart rate; FPG, fasting plasma glucose; BUN, blood urea nitrogen; eGFR, estimated glomerular filtration rate; hsCRP, high sensitivity C-reactive protein; UPCR, urine protein-to-creatinine ratio; Cr, creatinine. Adjusted OR and its CI were calculated using multivariate logistic regression analysis entering age, sex and variables with *P* < 0.05 in univariate logistic regression analysis as covariates.

**Figure 1:**
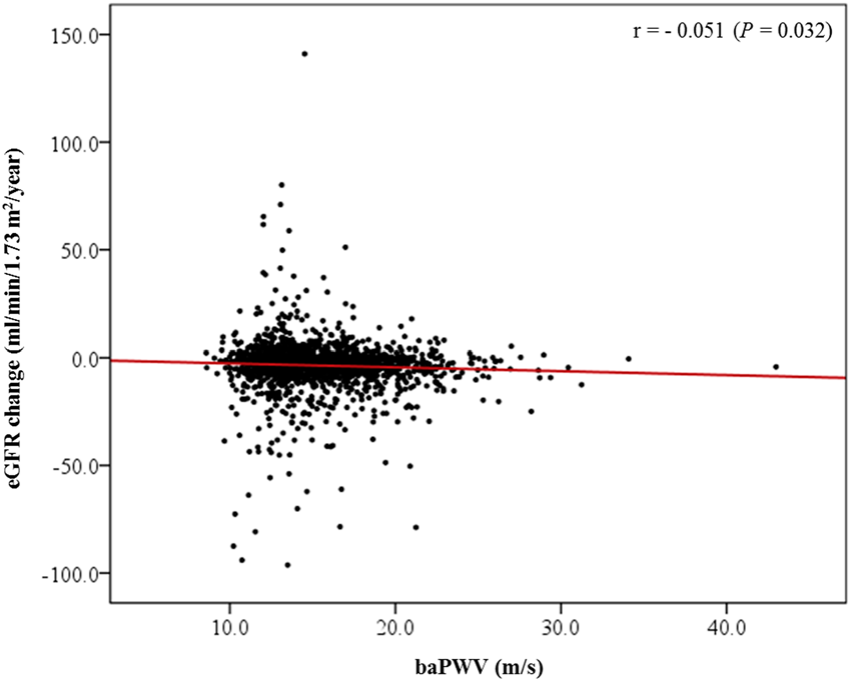
Scatter plot of the relationship between baPWV and changes in the eGFR. baPWV, brachial-ankle pulse wave velocity; eGFR, estimated glomerular filtration rate. The red line represents the regression line.

**Figure 2:**
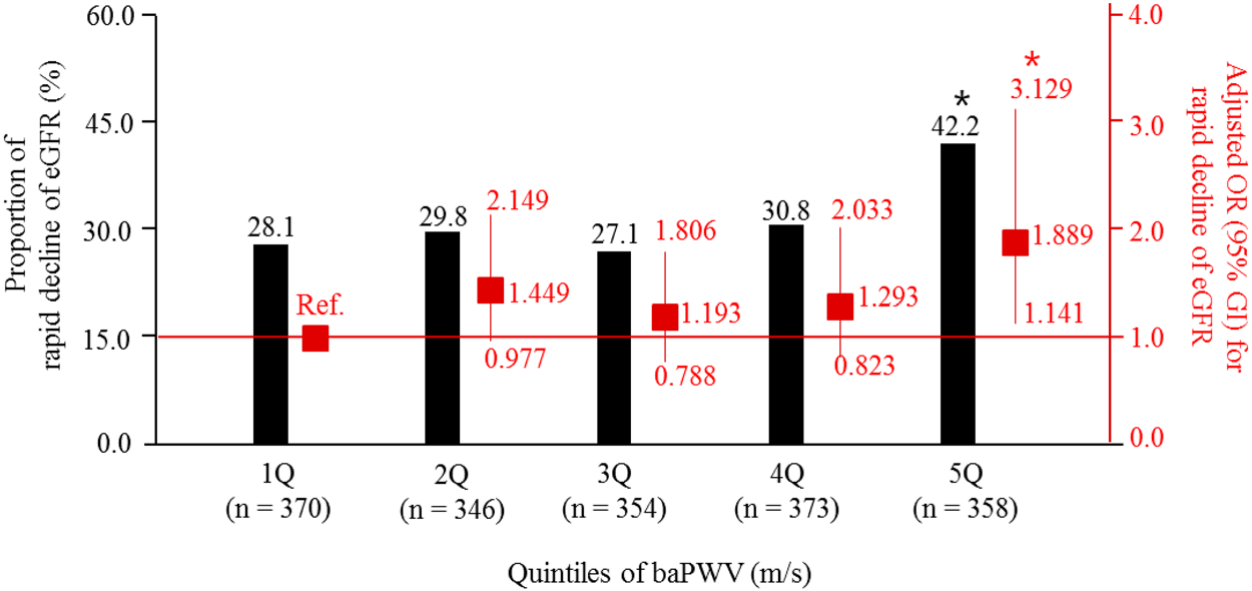
Risk for the rapid decline in the eGFR according to the status of the baPWV. baPWV, brachial-ankle pulse wave velocity; eGFR, estimated glomerular filtration rate; OR, odds ratio; CI, confidence interval. *P < 0.05 when compared with the 1Q of baPWV. The adjusted OR and its CI were calculated using a multivariate logistic regression analysis, into which age, sex, smoking pack-year, beta blocker use, diabetes, causes of chronic kidney diseases, heart rate, pulse pressure, serum levels of calcium and phosphorus, eGFR, bilirubin, albumin, cholesterol, hemoglobin, and logarithm of urine protein-to-creatinine ratio were entered.

**Figure 3:**
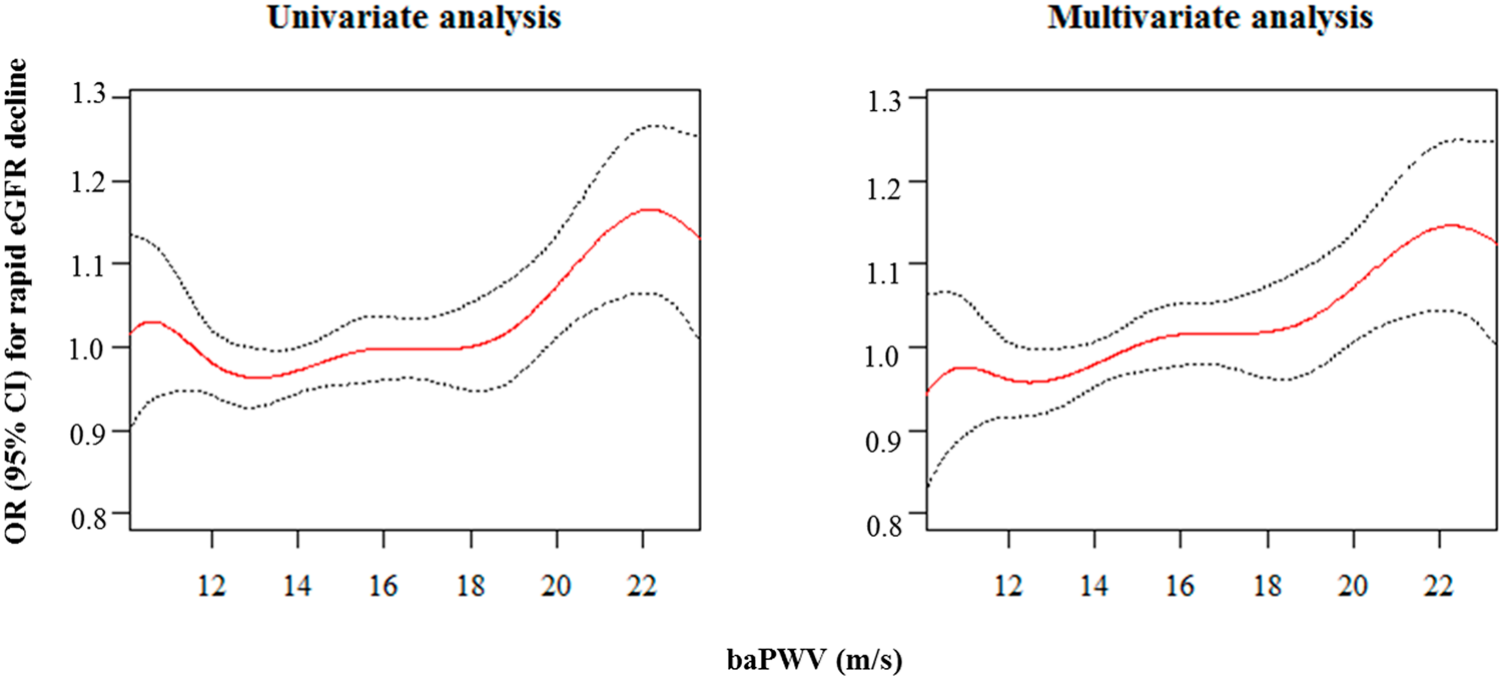
Penalized smoothing splines showing the relationship between the baPWV and the rapid decline in the eGFR. baPWV, brachial-ankle pulse wave velocity; eGFR, estimated glomerular filtration rate. OR, odds ratio; CI, confidence interval. The upper and lower 2.5% of the baPWV were truncated. The red line indicates the OR and the black dotted line indicates the 95% CI for which baPWV influences the rapid decline in the eGFR. In the multivariate analysis, the covariates were age, sex, smoking pack-year, beta blocker use, diabetes, causes of chronic kidney diseases, heart rate, pulse pressure, serum levels of calcium and phosphorus, eGFR, bilirubin, albumin, cholesterol, hemoglobin, and logarithm of urine protein-to-creatinine ratio.

We explored the association between the baPWV and the change of eGFR (ΔeGFR). Increased baPWV was inversely associated with the ΔeGFR (Fig. 1). In the multivariate linear regression analysis, every 1 m/s increase of the baPWV was associated with a ΔeGFR −0.229 ml/min/1.73 m^2^/year (95% CI = −0.436 to −0.022; *P* = 0.030) after adjusting for age, sex, systolic BP, pulse pressure, baseline eGFR, bilirubin, albumin, cholesterol, hemoglobin, and UPCR. We also analyzed the association between baPWV and the rapid decline of eGFR (Fig. 2). As the quintile of baPWV increased, the rate of rapid eGFR decline increased. In the multivariate logistic regression analysis entering age, sex, smoking pack-year, beta blocker use, diabetes, causes of CKD, heart rate, pulse pressure, serum levels of calcium and phosphorus, eGFR, logarithm of UPCR, bilirubin, albumin, cholesterol, and hemoglobin as covariates, the odds for a rapid decline in the eGFR were 1.9 times higher in the 5Q of the baPWV than in the 1Q of the baPWV (95% CI = 1.141–3.129; *P* = 0.013), suggesting that there was a non-linear association between the baPWV and a rapid decline in the eGFR. This non-linear association was confirmed in the penalized smoothing spline analysis, which was still unidirectional (Fig. 3).

**Figure 4:**
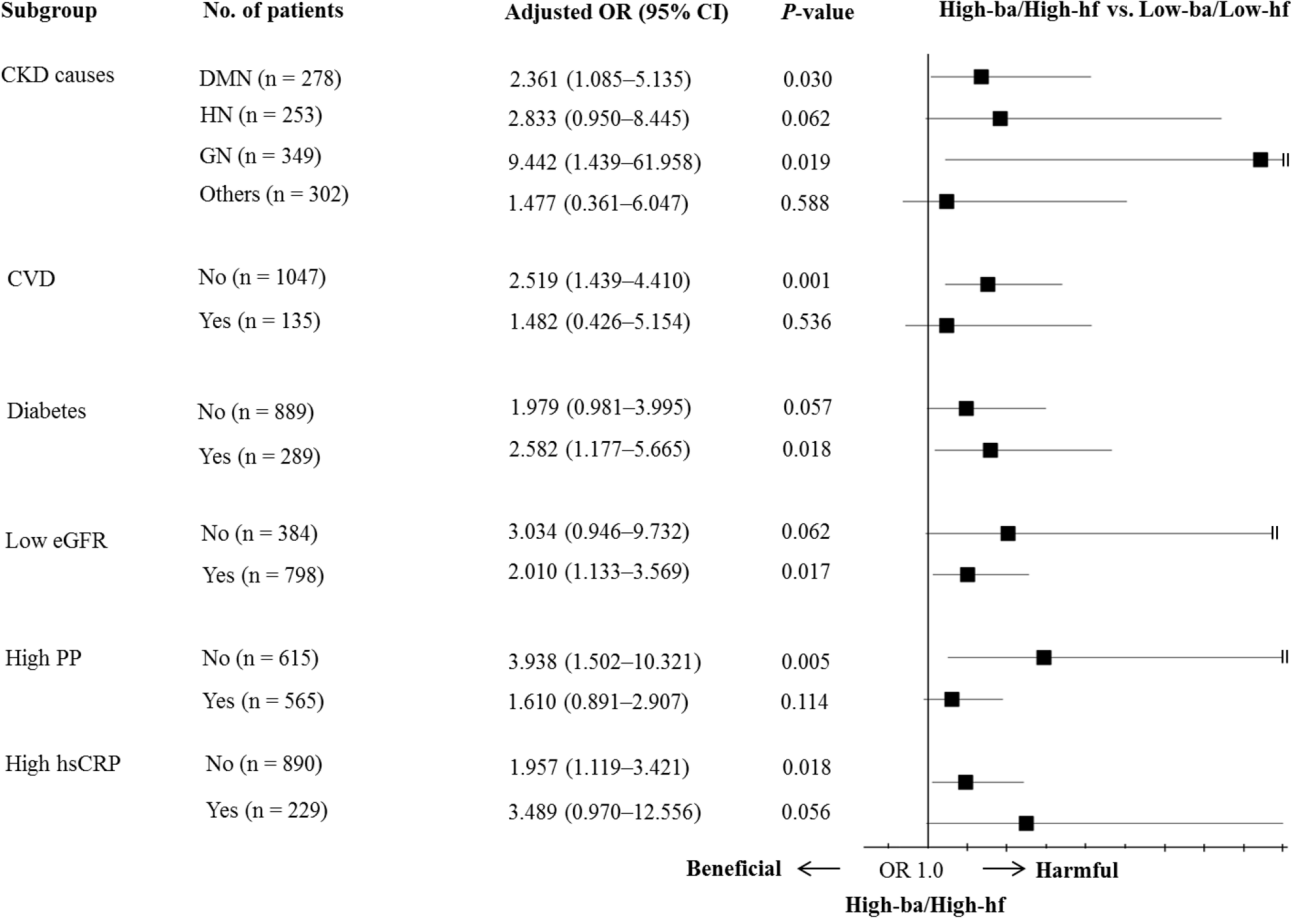
Subgroup analysis for the OR of both high baPWV and hfPWV (High-ba/High-hf) vs. both low baPWV and hfPWV (Low-ba/Low-hf) for a rapid eGFR decline. baPWV, brachial-ankle pulse wave velocity; hfPWV, heart-femoral pulse wave velocity; OR, odds ratio; CI, confidence interval; CKD, chronic kidney disease; CVD, cardiovascular disease; eGFR, estimated glomerular filtration rate; PP, pulse pressure. OR and its 95% CI were calculated using multivariate logistic analysis, entering into age, sex, diabetes, causes of CKD, PP, eGFR, logarithm of urine protein-to-creatinine ratio, albumin, cholesterol, hemoglobin, serum calcium level, and beta blocker use as covariates. When one covariate was chosen as a subgroup, it was omitted from the model.

To identify if there was a relationship between peripheral and central PWV, we compared the association of the baPWV and heart-femoral PWV (hfPWV) with a rapid decline in the eGFR in a subset of 1182 participants with both baPWV and hfPWV (Table 3). In the univariate logistic analysis, both the baPWV and hfPWV were associated with a rapid decline in the eGFR. However, in the multivariate logistic analysis, only the baPWV was associated with a rapid decline in the eGFR. Because there was a significant interaction between the hfPWV and baPWV and a rapid decline in the eGFR (beta 0.013; *P* = 0.035), we created four combined groups according to the status of baPWV and hfPWV: the low-ba/low-hf group (n = 860, 72.8%), high-ba/low-hf group (n = 87, 7.4%), low-ba/high-hf group (n = 106, 9.0%) and high-ba/high-hf group (n = 129, 10.9%). Participants in the high-ba/high-hf group had 2.1 times higher odds for having a rapid decline in the eGFR than those in the low-ba/low-hf group (*P* = 0.003) after adjusting for age, sex, diabetes, causes of CKD, pulse pressure, eGFR, logarithm of UPCR, albumin, cholesterol, hemoglobin, serum calcium levels, and beta blocker use. However, the odds for a rapid decline in the eGFR of participants in the high-ba/low-hf and low-ba/high-hf groups were not different from that of participants in the low-ba/low-hf group. In the subgroup analysis (Fig. 4), the odds for a rapid eGFR decline in participants with high-ba/high-hf were significantly higher in patients with diabetic nephropathy, glomerulonephritis, diabetes, and low eGFR, and in patients without CV disease, high pulse pressure, and high hsCRP.

**Table 3:** Risk of rapid decline of eGFR according to the status of baPWV and hfPWV in subset with both baPWV and hfPWV data.

Total (n = 1182)	Univariate		Multivariate	
	OR (95% CI)	*P*	Adjusted OR (95% CI)	*P*
baPWV (m/s)	1.061 (1.022–1.101)	0.002	1.084 (1.030–1.141)	0.002
hfPWV (m/s)	1.062 (1.014–1.112)	0.010	1.029 (0.961–1.103)	0.408
High-baPWV (yes vs. no)	1.770 (1.305–2.401)	<0.001	1.872 (1.269–2.761)	0.002
High-hfPWV (yes vs. no)	1.642 (1.220–2.210)	0.001	1.389 (0.936–2.061)	0.103
Combined groups (vs. low-ba/low-hf)		0.001		0.015
high-ba/low-hf	1.360 (0.853–2.168)	0.197	1.670 (0.968–2.880)	0.065
low-ba/high-hf	1.220 (0.790–1.883)	0.370	1.072 (0.626–1.835)	0.801
high-ba/high-hf	2.177 (1.493–3.175)	<0.001	2.100 (1.280–3.447)	0.003

eGFR, estimated glomerular filtration rate; OR, odds ratio; CI, confidence interval; hfPWV, heart-femoral PWV; baPWV, brachial-ankle pulse wave velocity.

OR and its CI were calculated using logistic regression analysis. In multivariate analysis, covariates were age, sex and variables with *P* < 0.05 in univariate logistic regression analysis for the rapid decline of eGFR (diabetes, causes of chronic kidney diseases, pulse pressure, eGFR, logarithm of urine protein-to-creatinine ratio, albumin, cholesterol, hemoglobin, serum calcium level, and beta blocker use).

## Discussion

Although many previous studies have suggested that increased arterial stiffness predicts a decline of eGFR levels^4,5,14–16^, their results do not always agree^17–20^. These previous studies used cfPWV as a marker of arterial stiffness. However, because cfPWV mostly reflects central or aortic stiffness^8^, using a broader marker of arterial stiffness may have a different clinical importance on the progression of CKD. In this regard, baPWV, which is a more convenient and reproducible marker of arterial stiffness than cfPWV^11,13,30^ may be an important alternative, because baPWV correlates well with both central and peripheral arterial stiffness^31^. Su and Chen *et al*. reported that increased baPWV was associated with an increased risk of the decline in kidney function^21–24^. However, studies that evaluated the association between baPWV and the decline in kidney function have been insufficient; the results were positive in some^25–27^, but negative in others^28,29^. Therefore, we performed the current study to identify the effect of baPWV on the decline in the eGFR, particularly focusing on its rapid change.

We explored the factors that were associated with an increased baPWV. Among others, older age, causes of CKD, and increased systolic BP and heart rate were independently associated with an increased baPWV, which was similar to the results of previous studies^32,33^. Wang *et al*. reported that the eGFR was associated with decreased odds of a high baPWV (OR 0.974, *P* = 0.04) in 344 Chinese adults^34^. In their study, people with high baPWV showed higher urine albumin-to-creatinine ratio (7.52 mg/g) than those with normal baPWV (vs. 6.07 mg/g, *P* = 0.021), but the significance was not maintained in multivariate logistic regression analysis. These results were opposite from those of our study, in which the baseline eGFR was significantly associated with an increased baPWV in the univariate analysis, but not in the multivariate analysis. UPCR was associated with an increased baPWV in both the univariate and multivariate analyses. The participants in the study by Wang *et al*. were younger (median 37 years) than those in our study and had normal kidney function without overt proteinuria. Because different clinical settings might affect study results, subsequent studies are needed to confirm our results.

In this study, baPWV was inversely associated with the ΔeGFR, which was concordant with the results of previous studies^21,22,27^. However, previous researchers did not further evaluate the association between baPWV and a rapid decline in the eGFR. In our study, the odds of a patient being in the 5Q of baPWV with a rapid decline in the eGFR were 1.9 times higher than that of the 1Q of baPWV. The central arteries act as a buffer to dampen the pressure from the left ventricle, whereas the peripheral arteries absorb energy during systolic blood flow^6^. Since the relative contribution of central and peripheral arterial stiffness to the rapid decline of the eGFR has not been fully determined, we further compared the association between the hfPWV and baPWV and a rapid decline in the eGFR. In the analysis, only the baPWV was independently associated with a rapid decline in the eGFR, which suggested that central arterial stiffness alone was not sufficient to predict a rapid decline in the eGFR levels. However, this did not mean that central arterial stiffness was not an important determinant for the rapid decline in the eGFR, because a high baPWV was not associated with a rapid decline in the eGFR if it was not accompanied by a high hfPWV.

In the subgroup analysis, the odds of both high central and peripheral PWV for a rapid eGFR decline was evident in patients with diabetes, diabetic nephropathy, glomerulonephritis, and decreased kidney function, which are risky, but not yet established CV diseases. However, both high central and peripheral PWV was not associated with a rapid eGFR decline in patients with established CV disease which pulse pressure was already increased, and systemic inflammation was elevated. This signifies that high both peripheral and central PWV may reflect subclinical organ damage. Therefore, our results suggest that patients with both high peripheral and central PWV need to be treated more meticulously with special attention to the development of a rapid eGFR decline.

This study has several strengths. First, this was the largest study that examined the clinical significance of the effect of the baPWV on the rapid decline in the eGFR levels. Second, because the study design was elaborate, we did not miss major variables. Third, simultaneous measurement of the hfPWV and baPWV enabled us to analyze these factors’ contributions to the rapid decline of eGFR levels. The study also has several limitations. First, the follow-up period was relatively short. The ΔeGFR may be exaggerated. Therefore, a longer follow-up period may stabilize the ΔeGFR value. However, the concordance of our results with those of previous studies may partly compensate for this limitation. Second, we did not evaluate other PWV data such as the cfPWV, heart-carotid PWV and femoral-ankle PWV. Therefore, a more detailed analysis of the contribution of central and peripheral arterial stiffness to the rapid decline in the eGFR was not possible. Third, the association between baPWV and hfPWV and eGFR change was modest, which may be affected by the imprecision of the measurement method. Finally, the single ethnicity of the patients in this study limits our ability to generalize our findings.

In conclusion, an increased baPWV independently predicted a rapid decline in the eGFR levels. Although increased central PWV alone did not sufficiently lead to a rapid decline in the eGFR levels, an increased central PWV positively affected the association between the baPWV and the rapid decline in eGFR levels. This suggested that both central and peripheral arterial stiffness are important determinants of the rapid decline of eGFR. Future studies are needed to confirm the results of our study.

## Materials and Methods

### Participants

The KNOW-CKD is a multicenter, prospective cohort study in Korea of 2238 patients with non-dialysis CKD stages 1–5 who were enrolled between February 2011 and January 2016. The detailed design and methods of the KNOW-CKD were previously published (NCT01630486 at http://www.clinicaltrials.gov)^35^. The protocol of the KNOW-CKD adhered to the principles of the Declaration of Helsinki and was approved by the institutional review board at each participating hospital, including Seoul National University Hospital, Yonsei University Severance Hospital, Kangbuk Samsung Medical Center, Seoul St. Mary’s Hospital, Gil Hospital, Eulji Medical Center, Chonnam National University Hospital, and Busan Paik Hospital. Written informed consent was obtained from all subjects. CKD and its stages were defined using the Kidney Disease Improving Global Outcomes 2012 guidelines^36^.

Of the 2238 cohort subjects, 437 were excluded, including 257 with missing baPWV data and 180 with missing data on the change in eGFR. Therefore, this study included 1801 patients (Figure S1).

### Measurement of PWV

The baPWV was automatically generated using a VP-1000 analyzer (Collin Co., Komaki, Japan)^10^. This device is an extension version of a device measuring ankle-to-brachial index which records the electrocardiogram, phonocardiogram, and pressure waveforms. Pressure waveforms of the brachial and tibial arteries were recorded with plethysmographic and oscillometric pressure sensors using occlusion/sensing cuffs that were applied to both arms and both ankles. The time intervals between pressure waveforms of the brachial and tibial arteries (pulse transit time) were measured, and baPWV were automatically calculated by the VP-1000 analyzer using the following formula: baPWV = [L_a_-L_b_]/[pulse transit time] (cm/s), where L_b_ is 0.2195* patient’s height (cm) − 2.0734 and L_a_ is 0.8129* patient’s height (cm)+12.328^10^. The mean value of the right and left baPWV was used as the main study factor. In this study, we transformed the unit from cm/s to m/s.

In a subset of the participants (n = 1182), we also measured hfPWV, which is a marker of central stiffness, using a VP-2000 analyzer (Collin Co., Komaki, Japan)^31^. Electrocardiogram electrodes were placed on both wrists. The heart sounds S1 and S2 were detected by a microphone on the left edge of the sternum at the fourth rib cage. The time intervals between S2 and the notch of the carotid pulse wave (Thc), and between the carotid and femoral artery pulse waves (Tcf) were measured at the left carotid and femoral arteries by the VP-2000 analyzer. The sum of Thc and Tcf gives the time required for pulse waves to travel from the heart (aortic orifice) to the femoral artery (Thf). The hfPWV was calculated using the following formula: hfPWV = Lhf/(Thc + Tcf), where Lhf is the distance from the heart to the femoral artery^37^.

The PWVs of the first (1Q), second (2Q), third (3Q), fourth (4Q), and fifth quintiles (5Q) were <12.6 m/s, 12.6–13.9 m/s, 13.9–15.4 m/s, 15.4–17.7 m/s, and ≥17.7 m/s, respectively, for baPWV and <8.1 m/s, 8.1–9.1 m/s, 9.1–10.2 m/s, 10.2–11.9 m/s, and ≥11.9 m/s, respectively, for hfPWV. We defined high baPWV and hfPWV as a PWV value that was in the highest quintile of each PWV group (baPWV ≥17.7 m/s and hfPWV ≥11.9 m/s).

### Rapid decline of eGFR

The serum creatinine concentrations were measured at a central laboratory (LabGenomics, Seongnam-si, Korea) using an Isotope Dilution Mass Spectrometry-calibrated Jaffe method. eGFR was calculated using the Modification of Diet in Renal Disease study formula^38^. At least two eGFR measurements that were taken 6 months apart were required to estimate the ΔeGFR. For each individual, the ΔeGFR was determined with a regression coefficient using a least-square linear regression analysis, with eGFR as a function of time in years, applied to all eGFR values that were obtained during the follow-up period in units of ml/min/1.73 m^2^/year. The main study outcome was the rapid decline of the eGFR, which was defined as a ΔeGFR <−5 ml/min/1.73 m^2^/year^39^. Mean follow-up time of ΔeGFR was 2.2 years.

### Other measurements and definitions

Clinical data, including detailed demographic information and baseline laboratory results, were extracted from the electronic data management system (PhactaX). When measuring baPWV, the VP-1000 device automatically measured systolic and diastolic blood pressure (BP) and heart rate. The difference between systolic and diastolic BP was pulse pressure. Hypertension was defined as systolic BP ≥ 140 mm Hg or diastolic BP ≥ 90 mmHg or treatment with anti-hypertensive drugs. Diabetes was defined as fasting glucose ≥126 mg/dl, or treatment with insulin or oral anti-diabetic drugs. Low eGFR was defined as eGFR <60 ml/min/1.73 m^2^. High pulse pressure and hsCRP were defined as above median values (50 mmHg in pulse pressure and 20 nmol/l in hsCRP). CV disease was defined as a physician diagnosis of cerebrovascular accidents, myocardial infarction, congestive heart failure, or peripheral arterial diseases. Body mass index was calculated as weight (kg) per square meter of height (m^2^). CV events were defined as hospitalization due to myocardial infarction (fatal and nonfatal), coronary revascularization, and stroke and new onset or aggravation of congestive heart failure.

### Statistical analysis

The distributions of continuous variables were evaluated using histograms and Q-Q plots. Two variables, hsCRP and the UPCR, were not normally distributed. Normally distributed continuous variables are expressed as the mean ± standard deviation, non-normally distributed continuous variables are expressed as the median (interquartile range), and categorical variables are shown as percentages. The *P*-trend was analyzed for normally distributed continuous variables using a linear-term of one-way analysis of variance (ANOVA), for non-normally distributed continuous variables using Jonckheere-Terpstra tests, and for categorical variables using a linear-by-linear association. Differences were analyzed using Bonferroni post-hoc analysis of a one-way ANOVA for normally distributed continuous variables, Mann-Whitney U tests for non-normally distributed continuous variables, and chi-square tests for categorical variables. The logarithms of hsCRP and UPCR were utilized in regression analyses. To assess linearity, we analyzed the regression coefficient (β) and 95% confidence interval (CI) with a linear regression analysis. The odds ratio (OR) and its 95% CI were calculated with a logistic regression analysis. A *P*-value of <0.05 was considered statistically significant. In the multivariate analysis, age, sex, and variables with statistical significance for the outcome variables that were determined in the univariate analysis were chosen as covariates. The interaction was analyzed by adding interaction-term in the regression modeling. The relationship between baPWV and the rapid decline of the eGFR was plotted using the penalized smoothing spline method, using the “pspline” package in R Statistics (version 3.03). All analyses, unless otherwise specified, were performed using SPSS Version 22 (IBM Corp., Armonk, NY).

### Data availability

Available as supplementary material when accepted.

## Electronic supplementary material

Supplementary Information
