# Supplementary material for: Relationship between brachial-ankle and heart-femoral pulse wave velocities and the rapid decline of kidney function
Source: Sci Rep. 2018 Jan 16;8:821. doi: 10.1038/s41598-018-19334-w (PMC5770427; doi:10.1038/s41598-018-19334-w)
Supplement: Supplementary file 1 — Supplementary Information [file 41598_2018_19334_MOESM1_ESM.pdf]

Relationship between brachial-ankle and heart-femoral pulse wave velocities and the rapid decline of kidney function

Sung Woo Lee[1,2], Seung Hyeok Han[3], Tae Hyun Yoo[3], Wookyung Chung[4], Sue K. Park[5,6,7], Dong Wan Chae[8], Curie Ahn[8], and *Kook-Hwan Oh[8]

[1]*Department of Internal Medicine, Seoul National University Postgraduate School, Seoul, Korea*

[2]*Department of Internal Medicine, Eulji University, Eulji General Hospital, Seoul, Korea*

[3]*Department of Internal Medicine, Yonsei University College of Medicine, Seoul, Korea*

[4]*Department of Internal Medicine, Gachon University, Gil Hospital, Incheon, Korea*

[5]*Department of Preventive Medicine, Seoul National University College of Medicine, S eoul, Korea*

[6]*Cancer Research Institute, Seoul National University, Seoul, Korea*

[7]*Department of Biomedical Science, Seoul National University Graduate School, Seoul, Korea*

[8]*Department of Internal Medicine, Seoul National University College of Medicine, Seoul, Korea*

***Correspondence to: Kook-Hwan Oh**

e-mail : ohchris@hanmail.net

Tel) 82-2-2072-0776

Fax) 82-2-741-4876

Address) Department of Internal Medicine, Seoul National University Hospital 101 *Daehak-ro, Chong No Gu* 03080, Seoul, Korea

Figure S1. Flowchart of the patients' selection. baPWV, brachial-ankle pulse wave velocity; eGFR, estimated glomerular filtration rate.

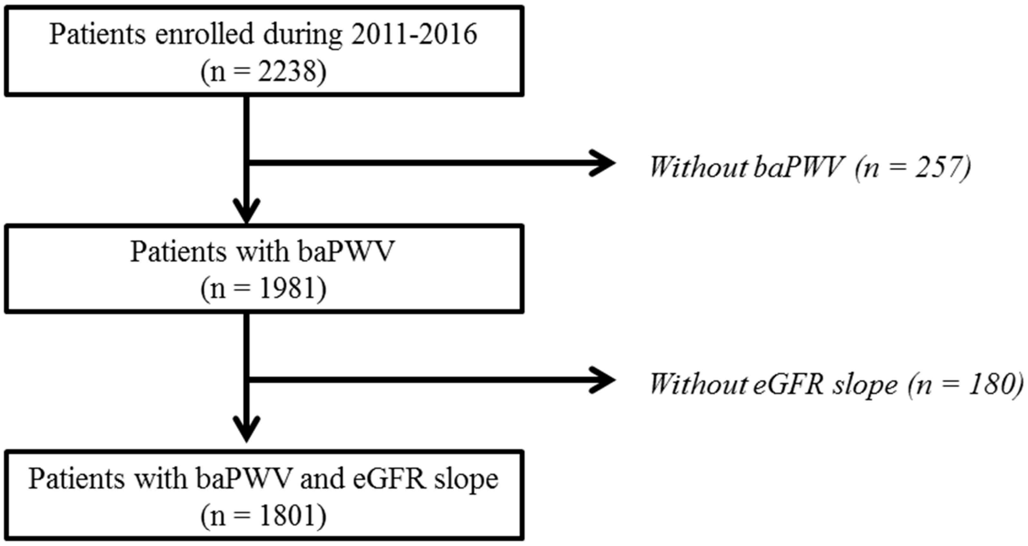

Table S1. Clinical characteristics of study population according to CKD stages

	Stage 1 (n = 230)	Stage 2 (n = 350)	Stage 3 (n = 721)	Stage 4 (n = 405)	Stage 5 (n = 89)	*P*
Age (years)	44.1 ± 11.7	50.7 ± 11.6*	56.5 ± 11.2*	57.5 ± 10.9*	56.1 ± 11.8*	<0.001
Male sex (%)	47.8	66.1*	65.0*	59.2*	42.7	0.938
Alcohol drinking (%)	56.6	55.6	44.0*	34.5*	23.8*	<0.001
Smoking (Pack-year)	7.0 ± 15.0	10.7 ± 16.9	12.9 ± 18.8*	14.2 ± 20.7*	8.7 ± 17.2	0.001
Hypertension (%)	89.6	95.2*	96.7*	97.3*	96.6*	<0.001
RAS inhibitor (%)	79.6	88.6*	88.5*	86.8*	86.5*	0.078
Beta blocker (%)	14.3	17.1	25.9*	32.5*	31.5*	<0.001
CCB (%)	23.0	33.9*	45.5*	54.3*	60.7*	<0.001
Diuretics (%)	11.7	17.9*	32.3*	44.7*	51.7	<0.001
Diabetes (%)	11.5	13.5	28.9*	38.8*	37.1*	<0.001
CVD (%)	3.5	5.1	12.5*	14.7*	9.0*	<0.001
Cause of CKD						
DMN (%)	7.0	13.1*	26.0*	39.6*	36.0*	<0.001
HN (%)	8.3	16.5*	24.2*	22.5*	15.7*	<0.001
GN (%)	45.7	36.8*	31.3*	24.2*	31.5*	<0.001

Others (%)	39.1	33.6	18.4*	13.7*	16.9*	<0.001
SBP (mm Hg)	126.4 ± 14.4	127.1 ± 15.2	128.3 ± 15.9	130.4 ± 17.3*	131.4 ± 19.1	<0.001
DBP (mm Hg)	77.6 ± 10.4	77.8 ± 11.2	76.5 ± 10.6	76.6 ± 12.2	75.6 ± 10.7	0.050
PP (mmHg)	48.8 ± 10.5	49.3 ± 11.6	51.8 ± 13.1*	53.8 ± 13*	55.8 ± 15.2*	<0.001
HR (counts/min)	73.4 ± 13.1	73.9 ± 13.0	72.8 ± 12.7	73.7 ± 13.0	71.8 ± 12.8	0.491
baPWV (m/s)	13.3 ± 2.0	14.4 ± 2.8*	15.6 ± 3.3*	16.4 ± 3.8*	16.0 ± 3.4*	<0.001
hfPWV (m/s)	8.5 ± 1.5	9.4 ± 2.1*	10.4 ± 2.6*	11.0 ± 3.0*	10.8 ± 2.8*	<0.001
BMI (kg/m^2)	24.1 ± 3.7	24.5 ± 3.4	24.7 ± 3.2*	24.5 ± 3.4*	24.3 ± 3.4	0.338
FPG (mmol/l)	5.7 ± 1.3	5.9 ± 1.7	6.3 ± 2.3	6.3 ± 2.5	6.4 ± 2.6	<0.001
BUN (mmol/l)	4.8 ± 1.3	6.1 ± 1.8*	8.9 ± 2.8*	14.5 ± 4.4*	21 ± 6.4*	<0.001
Calcium (mmol/l)	2.31 ± 0.11	2.32 ± 0.10	2.30 ± 0.12	2.24 ± 0.12*	2.15 ± 0.18*	<0.001
Phosphorus (mmol/l)	1.14 ± 0.17	1.12 ± 0.18	1.15 ± 0.18	1.26 ± 0.21*	1.47 ± 0.19*	<0.001
Cr (μmol/l)	61.8 ± 12.3	88.7 ± 15.1*	137.9 ± 28.5*	236.5 ± 52.6*	382.7 ± 75.4*	<0.001
eGFR ($ml/min/1.73m^2$)	110.1 ± 19.8	73.3 ± 8.6*	43.7 ± 8.7*	23.0 ± 4.4*	12.5 ± 1.7*	<0.001
Bilirubin (μmol/l)	14.6 ± 6.2	13.4 ± 5.5*	11.6 ± 4.6*	9.0 ± 3.4*	8.1 ± 2.8*	<0.001

Albumin (g/l)	43.2 ± 3.9	43.0 ± 3.7	42.0 ± 4*	40.5 ± 4.4*	40.5 ± 4.2*	<0.001
Cholesterol (mmol/l)	4.8 ± 1.0	4.7 ± 0.9	4.5 ± 1.0*	4.4 ± 1.1*	4.4 ± 1.0*	<0.001
Hemoglobin (g/dl)	14.0 ± 1.5	14.1 ± 1.7	13.0 ± 1.9*	11.5 ± 1.5*	10.5 ± 1.2*	<0.001
Ln-hsCRP (nmol/l)	1.4 ± 1.3	1.8 ± 1.3*	1.8 ± 1.4*	2.1 ± 1.4*	1.8 ± 1.3	<0.001
Ln-UPCR (g/g Cr)	-1.5 ± 1.5	-1.5 ± 1.5	-0.8 ± 1.5*	-0.1 ± 1.3*	0.1 ± 1.2*	<0.001

RAS, renin angiotensin-aldosterone system; CCB, calcium channel blocker; CVD, cardiovascular disease; baPWV, brachial-ankle pulse wave velocity; CKD, chronic kidney disease; DMN, diabetic nephropathy; HN, hypertensive nephropathy; GN, glomerulonephritis; SBP, systolic blood pressure; DBP, diastolic blood pressure; PP, pulse pressure; hfPWV, heart-femoral pulse wave velocity; BMI, body mass index; FPG, fasting plasma glucose; BUN, blood urea nitrogen; Cr, creatinine; eGFR, estimated glomerular filtration rate; hsCRP, high sensitivity C-reactive protein; UPCR, urine protein-to-creatinine ratio.

Values are expressed as mean ± standard deviation for continuous variables and percentage for categorical variables. *P*-trend was analyzed by linear-term of one-way ANOVA for continuous variables and a linear-by-linear association for categorical variables. Except for hfPWV (619/1801, 34.4%) and smoking year (286/1801, 15.9%), missing rate of all above variables was below 8.9%.

* meant $P < 0.05$ when compared to CKD stage 1 by using Bonferroni post-hoc analysis of one-way ANOVA for continuous variables and chi-square test for categorical variables.
